# Activation of the AIM2 Receptor in Circulating Cells of Post-COVID-19 Patients With Signs of Lung Fibrosis Is Associated With the Release of IL-1α, IFN-α and TGF-β

**DOI:** 10.3389/fimmu.2022.934264

**Published:** 2022-06-29

**Authors:** Chiara Colarusso, Michela Terlizzi, Angelantonio Maglio, Antonio Molino, Claudio Candia, Carolina Vitale, Philip M. Hansbro, Alessandro Vatrella, Aldo Pinto, Rosalinda Sorrentino

**Affiliations:** ^1^ Department of Pharmacy, University of Salerno, Fisciano, Italy; ^2^ Department of Medicine and Surgery, University of Salerno, Baronissi, Italy; ^3^ Department of Respiratory Medicine, Respiratory Division, University of Naples Federico II, Naples, Italy; ^4^ Centre for Inflammation, School of Life Sciences, Faculty of Science, Centenary Institute and University of Technology Sydney, Sydney, NSW, Australia

**Keywords:** SARS–CoV–2, AIM2 inflammasome, post-COVID-19, cytokines, post-COVID-19 lung fibrosis

## Abstract

Severe acute respiratory syndrome-coronavirus-2 (SARS‐CoV‐2), responsible for COVID-19, has caused a global pandemic. Observational studies revealed a condition, herein called as Long-COVID syndrome (PC), that affects both moderately and severely infected patients, reducing quality-of-life. The mechanism/s underlying the onset of fibrotic-like changes in PC are still not well defined. The goal of this study was to understand the involvement of the Absent in melanoma-2 (AIM2) inflammasome in PC-associated lung fibrosis-like changes revealed by chest CT scans. Peripheral blood mononuclear cells (PBMCs) obtained from PC patients who did not develop signs of lung fibrosis were not responsive to AIM2 activation by Poly dA:dT. In sharp contrast, PBMCs from PC patients with signs of lung fibrosis were highly responsive to AIM2 activation, which induced the release of IL-1α, IFN-α and TGF-β. The recognition of Poly dA:dT was not due to the activation of cyclic GMP-AMP (cGAMP) synthase, a stimulator of interferon response (cGAS-STING) pathways, implying a role for AIM2 in PC conditions. The release of IFN-α was caspase-1- and caspase-4-dependent when AIM2 was triggered. Instead, the release of pro-inflammatory IL-1α and pro-fibrogenic TGF-β were inflammasome independent because the inhibition of caspase-1 and caspase-4 did not alter the levels of the two cytokines. Moreover, the responsiveness of AIM2 correlated with higher expression of the receptor in circulating CD14+ cells in PBMCs from patients with signs of lung fibrosis.

## Introduction

Severe acute respiratory syndrome-coronavirus-2 (SARS‐CoV‐2), the virus responsible for COVID-19, has so far infected millions of people (1) and caused more than 5.9 million deaths worldwide (https://covid19.who.int/). Symptoms associated with COVID-19 are defined as mild and/or moderate when dry cough, fatigue, anosmia and fever occur. Severe symptoms are related to respiratory failure (2), which requires hospitalization for oxygen supplementation and/or invasive mechanical ventilation and, in some cases, intensive care (3). Although most COVID-19 patients completely recover, about one-third experience prolonged symptoms that last for more than 4 weeks after recovery from acute infection, a condition termed as post-COVID-19 (PC) syndrome or long COVID (4).

PC syndrome is recognized as a serious consequence of SARS-CoV-2 infection and involves persistent physical, medical and cognitive sequelae that vary from patient to patient. It is characterized by symptoms such as fatigue, dyspnea, cognitive impairment, cough, myalgia, and arthralgia after infection resolution (5). Observational studies have highlighted that among PC patients 90% experience respiratory problems and lung tissue damage, which may lead to the establishment of fibrotic lung disease (6, 7). Indeed, it is estimated that there is a risk of 2-6% of developing lung fibrosis in patients who were affected by moderate and severe COVID-19 (1). To date, although the mechanism/s underlying the onset of fibrotic-like changes in PC condition are not well defined, clinical evidence shows that PC-associated lung fibrosis could lead to worsened quality-of-life and long-term disability from the progressive lung damage associated with the impairment of gas exchange. Recent studies do show that there are similarities between fibrosis in COVID-19 and other conditions (7, 8). Since PC is becoming more frequent, increasing the understanding of the molecular mechanism/s underlying this phenomenon would be valuable in identifying 1. biomarkers to facilitate the prevention of the development of lung fibrosis after COVID-19 recovery, and 2. potential therapeutic targets and drugs to be repurposed for treatment.

In our previous studies, we showed that absent in melanoma 2 (AIM2) inflammasome, a multimeric protein complex, was responsible for the release of IL-1α and TGF-β by peripheral blood mononuclear cells (PBMCs) of idiopathic pulmonary fibrosis (IPF) patients (9). We also showed roles in neutrophilic inflammation in experimental chronic obstructive pulmonary disease (COPD) (10). Interestingly, PC patients with ground-glass opacities (GGO) revealed by chest CT scan were characterized by higher plasma levels of IL-1α and TGF-β, compared to healthy subjects increasing the relative risk (RR=2.8) of fibrotic-like changes (11).

Although, some evidence has demonstrated that SARS-CoV-2 directly or indirectly activates inflammasome/s (12), the goal of this study was to understand whether any links between AIM2 and PC syndrome exist. The inflammasome is a multiprotein complex that, once activated, comprises the assembly of an upstream receptor, such as Nod-like receptors (NLRs) or hematopoietic interferon-inducible nuclear proteins with a 200-amino-acid repeat (HIN-200) family receptor (i.e., AIM2). It also comprises downstream proteins, such as the adaptor apoptosis-associated speck-like protein containing a CARD (ASC) (13, 14). ASC binds, in turn, to the caspase-1 that autocleaves and induces the release of the pleiotropic IL-1 family cytokines (i.e. IL-1β and IL-18) as well as the induction (in some circumstances and cell types) of pyroptosis (13). The non-structural protein 6 (NSP6) of SARS-CoV-2 was shown to target NOD-like receptor family pyrin domain-containing 3 (NLRP3)/ASC-dependent caspase-1 activation, IL-1β/-18 maturation, and pyroptosis of lung epithelial cells (15). In support of this, specific inhibition of the NLRP3 inflammasome by MCC950 alleviated excessive lung inflammation and thus COVID-19-like pathology in human Angiotensin-converting enzyme 2 (ACE2) transgenic mice infected with SARS-CoV-2 (16). On the other hand, the use of colchicine that can inhibit NLRP3, had no clinical benefit in patients with COVID-19 (17). The role of inflammasomes in PC is unknown.

Here, we assessed the role of inflammasomes in PC patients, focusing on the role of AIM2 receptor to define the cellular source of cytokines, which likely contribute to the development of lung fibrosis in susceptible PC patients.

## Materials and Methods

### Human Samples

The experimental protocol was performed according to the guidelines and regulations provided by the Ethical Committee Board of the Monaldi-Azienda Ospedaliera (AORN)-Ospedale dei Colli Hospital in Naples (Italy protocol n.422/2017, 729/2020). All participants involved in this study were recruited at this Hospital and gave informed consent according to the guidelines of the Review Board of the hospital. Peripheral blood samples were collected from healthy vaccinated (HV; n=14) subjects and PC patients (n=48) after 1–3 months of negative oral-pharyngeal swabs to detect SARS-CoV-2 infection (mRNA-based analysis). HVs did not have any pathology or haematological alterations. Based on the presence of GGO and reticular/fibrotic areas on high resolution CT (HRCT) scans of the lungs, and to evaluate functional and clinical parameters, PC patients were stratified as with (n=28) or without (n=20) fibrosis-like changes. All subjects/patients had no previous history of allergic diseases or chronic respiratory conditions. The age of enrolled HV subjects and PC patients had a mean of 30 ± 10 and 55 ± 10 years old, respectively. Blood samples were collected and used within 24 hours.

### Isolation of Human PBMCs

PBMCs were isolated using Ficoll gradients as previously reported (18). Briefly, blood (5 mL) was mixed with Roswell Park Memorial Institute (RPMI) 1640 cell media (5 mL) supplemented with antibiotics (1% Penicillin-Streptomycin) and gently layered on the top of Ficoll medium. Samples were centrifuged (1,125 x*g*, 20 min) and PBMC layers collected, diluted with RPMI cell medium and centrifuged (753 x*g*, for 20 min) to remove the remaining Ficoll solution. Platelets were separated by centrifugation (149 x*g*, 10 min) and PBMCs were collected.

### PBMC Culture

Freshly isolated PBMCs were cultured in RPMI media supplemented with 1% Penicillin-Streptomycin and 10% Fetal Bovine Serum (5% CO_2_, 37°C). PBMCs were seeded in 96-well plates (2×10^5^ cells/well) and treated at different time points (5 or 24 hours) with: an AIM2 inflammasome ligand Poly dA:dT (19) (1 μg/mL; Invivogen, Toulouse, France), a caspase-1 inhibitor Ac-YVAD-cmk (YVAD, 1 μg/mL; Sigma-Aldrich, Merck Life Science S.r.l., Milan, Italy), a caspase-4 inhibitor ICH-2 (20) (ICH2, 6.4 μg/ml; Sigma-Aldrich, Merck Life Science S.r.l., Milan, Italy), a human cyclic GMP-AMP (cGAMP) synthase (cGAS) inhibitor G140 (2 μg/ml; Invivogen, Toulouse, France), and an irreversible and selective inhibitor of STING H-151, (H151, 1 μg/mL; Invivogen, Toulouse, France). Concentrations of the above treatments were chosen from published data (9, 11, 18, 19, 21). In particular, the working concentration of the inhibitors Ac-YVAD-cmk and ICH2 inhibitors was used according to previous studies (9, 13, 18, 19). G140 was used at the concentration of 2 μg/ml because it was able to inhibit cGAS-induced cGAMP (data not shown), despite the datasheet reported IC50 which had too high concentration of DMSO that could alter cell viability (maximum accepted DMSO is 0.1%). H150 was used at the concentration reported by the manufacturer’s datasheet. 

### Chest CT Scans

Chest CT scans were performed 1–3 months after the first negative oral-pharyngeal swab. Patients were examined in the supine position, covering the area from the apex of the lung to the costophrenic angle with a scanning layer thickness and layer spacing of 0.5-2 mm (HRTC). Pulmonary involvement was measured by expert radiologists by applying a semi-quantitative scoring system to the pulmonary area involved in fibrosis (22). Each of the five lung lobes were visually scored from 0 to 5 as: 0, no involvement; 1, <5%, 2, 25%; 3, 26–49%; 4, 50–75%, and 5, >75% involvement. The total CT score was the sum of the individual lobar scores and ranged from 0 (no involvement) to 25 (maximum involvement) (23). In this study, we evaluated the presence of fibrotic or non-fibrotic patterns by applying a qualitative measure closely associated with the semi-quantitative score previously described. The presence of fibrotic pattern/s was considered in patients with a score >5.

### Cytokine Measurements

IL-1α, IFN-α, IFN-β, IL-18, IL-33 and IL-1β were evaluated in cell-free supernatants from PBMCs after 5 hours of treatment with inhibitors. TGF-β levels were analyzed after 24 hours of treatment. All were quantified using commercial ELISA kits following the manufacturer’s instructions (Diaclone SAS, France; Invitrogen, Thermo Fisher Scientific Inc, Vienna, Austria; R&D Systems, Bio-Techne, Minneapolis, USA).

### Flow Cytometry Analysis

AIM2 expression was analyzed using flow cytometry (BD FacsCalibur Milan, Italy) after the addition of the following antibodies to isolated PBMCs: CD14-PE, HLA-DR-PerCP and AIM2-APC (eBioscience, CA, United States). PBMCs were stained for cell surface CD14 and HLA-DR, fixed and then permeabilized using BD Cytofix/Cytoperm solutions before adding anti-AIM2.

### Real-Time Polymerase Chain Reaction (RT-PCR)

AIM2 gene expression was measured using RT-PCR in RNA isolated from untreated PBMCs (10^7^ cells/well). Total RNA was isolated from cells using the RNA extraction kits according to the manufacturer’s instructions (Qiagen, Milan, Italy). Reverse transcription was performed using first-strand cDNA synthesis kit (Qiagen, Milan, Italy) followed by PCR. Thermal cycling conditions were: 5 min at 95°C, followed by 45 cycles of 30 s at 95°C (denaturation), 30 s at 60°C (annealing), 30 s at 72°C (elongation) for AIM2 and for β-actin;

Primer pairs were:

AIM2: Forward 5′-GACGAGTTTAATATTGCCACAGG-3′
Reverse 5′-TCCTGAAGACGTTTTGCCAAA-3′
β-actin: Forward 5′-ACTCTTCCAGCCTTCCTTCC-3′
Reverse 5′-CGTACAGGTCTTTGCGGATG-3′

### Agarose Gel Electrophoresis

Agarose gel electrophoresis was performed to confirm the size of RT-PCR products. The amplified RT-PCR products of the AIM2 gene (expected size: 158 bp) were subjected to 2% agarose gel electrophoresis (under 90 V for 1-2 hours) and stained with RedSafe Nucleic Acid Staining Solution (iNtRON Biotechnology, Inc., Korea). For the determination of fragments size, a 25 bp DNA ladder was used (Promega Corporation, USA).

### Statistical Analysis

**Figure 1:**
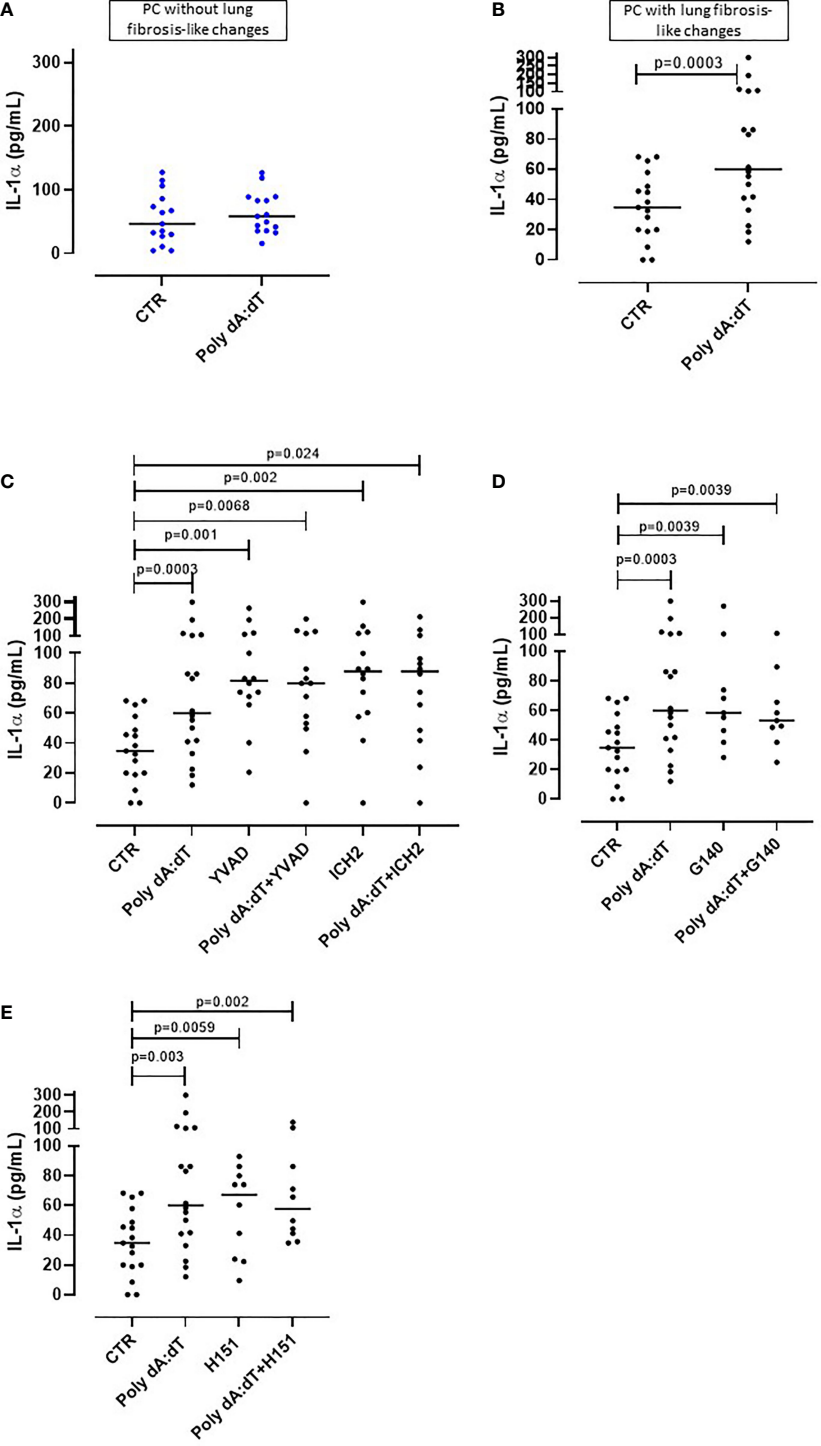
AIM2 receptor activation induced IL-1α release from PBMCs from Post-COVID-19 patients with fibrosis. PBMCs were stimulated with Poly dA:dT (1 μg/mL) for 5 hours. **(A)** PBMCs from Post-COVID-19 (PC) patients who did not develop lung fibrosis did not release IL-1α after AIM2 activation. **(B)** PBMCs from PC patients who developed lung fibrosis released greater amounts of IL-1α after AIM2 activation. **(C)** Inhibition of caspase-1 and caspase-4 with Ac-YVAD-cmk (YVAD, 1 μg/mL) and ICH2 (ICH2, 6,4 μg/ml), respectively, did not reduce the release of IL-1-α after Poly dA:dT stimulation. Inhibition of **(D)** cGAS with G140 (2 μg/ml) or **(E)** STING with H-151 (H151, 1 µg/mL), did not alter the release of IL-1α after Poly dA:dT stimulation. Data are reported as median and represented as scatter dot plots. Statistical analysis was performed using the Wilcoxon matched-pairs signed rank test.

**Figure 2:**
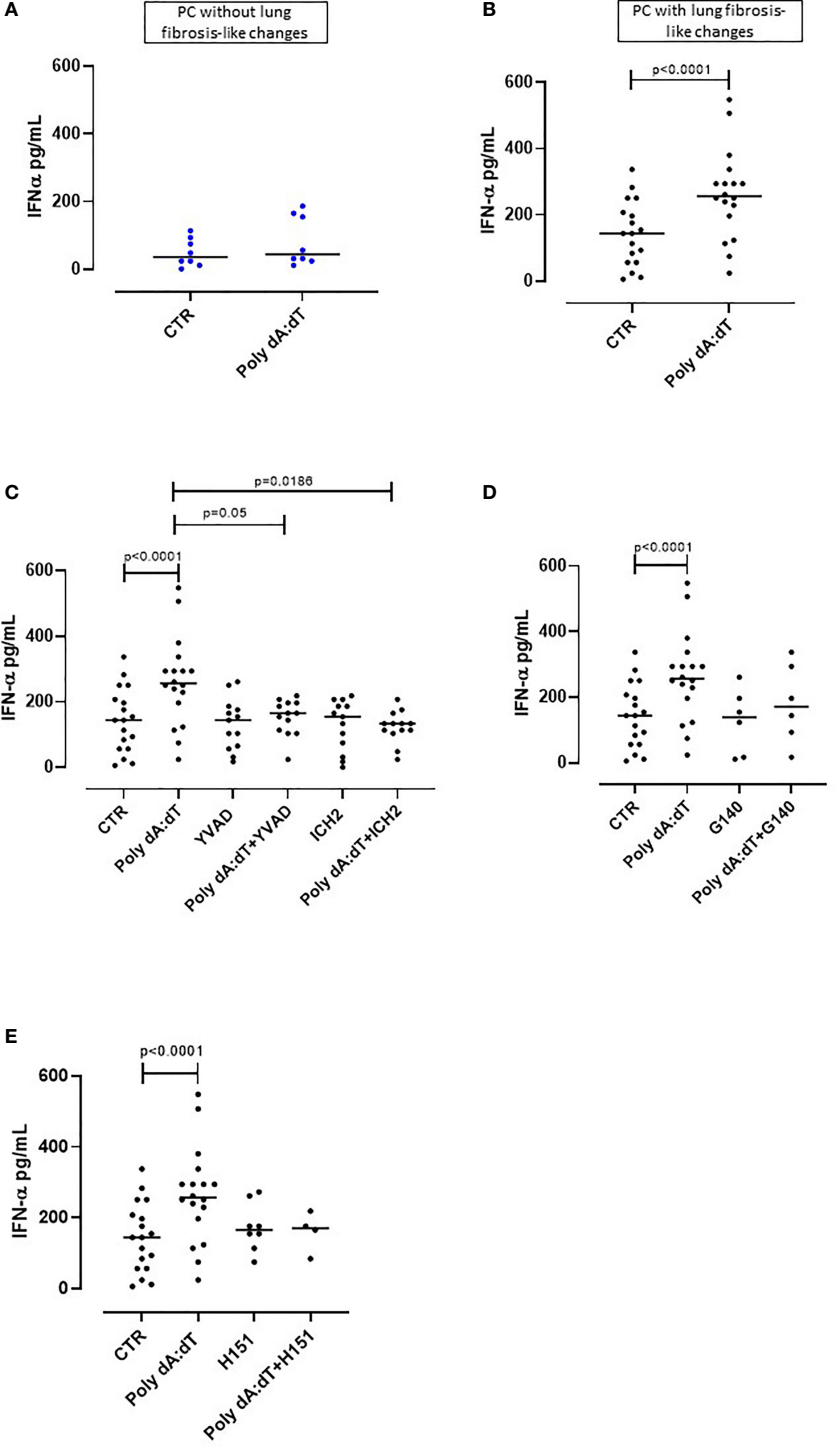
AIM2 inflammasome activation induced IFN-α release from PBMCs obtained from Post-COVID-19 patients with fibrosis. PBMCs were stimulated with Poly dA:dT (1 μg/mL) for 5 hours. **(A)** PBMCs from Post-COVID-19 (PC) patients who did not develop lung fibrosis did not release IFN-α after AIM2 activation. **(B)** PBMCs from PC patients who developed lung fibrosis significantly released IFN-α after AIM2 activation. **(C)** Inhibition of caspase-1 and caspase-4 with Ac-YVAD-cmk (YVAD, 1 μg/mL) and ICH2 (ICH2, 6,4 μg/ml), respectively, did not reduce the release of IFN-α after Poly dA:dT stimulation. Inhibition of **(D)** cGAS with G140 (2 μg/ml) or **(E)** STING with H-151 (H151, 1 µg/mL), did not alter the release of IFN-α after Poly dA:dT stimulation. Data are reported as median and represented as scatter dot plots. Statistical analysis was performed using the Wilcoxon matched-pairs signed rank test.

Data are reported as median and represented as scatter dot plots. Statistical differences were assessed with two-tailed Wilcoxon matched-pair t-test (**Figures 1**, **2**, **4** and **Supplementary Figure 2**) and Mann-Whitney U test (**Figure 5**). p values less than 0.05 were considered significant. Statistical analysis was performed by using GraphPad prism 9.3.1 version (San Diego, USA).

## Results

### Activation of AIM2 Inflammasome Induced the Release of IL-1α From PBMCs From PC Patients With Signs of Lung Fibrosis

To investigate the potential involvement of AIM2 inflammasome in PC syndrome, we stimulated PBMCs with Poly dA:dT (1 μg/mL), a synthetic cytosolic dsDNA AIM2 ligand. The addition of Poly dA:dT for 5 hours onto PBMCs from patients who did not develop PC syndrome did not increase the levels of IL-1α (**Figure 1A**, ctr: 55.45 ± 10.3 pg/ml vs PolydA:dT: 64.3 ± 8.45 pg/ml). In contrast, AIM2 stimulation in PBMCs from patients who presented lung fibrosis-like changes significantly increased (p=0.01) IL-1α levels (**Figure 1B**, ctr: 34.75 ± 5.42 pg/ml vs PolydA:dT: 81.6 ± 16.5 pg/ml). Notably, IL-1α release was observed in 18 out of 24 PC patients with lung fibrosis-like changes (75%) after AIM2 activation.

To understand and define the molecular mechanism/s associated with AIM2-dependent IL-1α release from lung fibrotic PC-derived PBMCs, we treated the cells with a caspase-1 inhibitor, Ac-YVAD-cmk (YVAD, 1 μg/mL), or a caspase-4 inhibitor ICH2 (ICH2, 6.4µg/ml) with or without Poly dA:dT challenge. The administration of either YVAD or ICH2 did not alter the release of IL-1α after Poly dA:dT challenge (**Figure 1C**), suggesting that IL-1α release was caspase-1- and caspase-4-independent. Since the cytosolic dsDNA Poly dA:dT can also be recognized by other cytosolic DNA sensors such as cGAS (cyclic-GMP-AMP synthase) (24), we treated cells with a specific inhibitor of human cGAS, G140 (2µg/ml) to define its involvement in AIM2-dependent signaling. Inhibiting cGAS did not alter IL-1α release after AIM2 activation, implying that cGAS is not involved in AIM2 signaling (**Figure 1D**). Furthermore, it has been described that the stimulator of interferon genes (STING) pathway that underpins cGAS activity can interfere with AIM2 activity (25). Thus, we inhibited STING with an irreversible and selective small molecule H151 (1 μg/mL) to rule out dysfunctional cGAS activity in our findings. STING inhibition did not alter AIM2-induced IL-1α release after PolydA:dT challenge (**Figure 1E**). Moreover, NLRP3 activation with LPS (0.1 μg/mL) and ATP (0.25 mg/ml) did not induce the release of IL-1α (data not shown).

Together these data provide strong evidence that AIM2 inflammasome activation induces IL-1α release from circulating immune cells of PC patients with signs of lung fibrosis, implying systemic inflammation.

### Activation of AIM2 Inflammasome Led to the Release of IFN-α From Fibrotic PC-Derived PBMCs That Was Caspase-1- and Caspase-4-Dependent

Type I interferons (IFN-I) are anti-viral cytokines that play a key role in SARS-CoV-2 infection (26). Although IFN-I initially have a protective function against virus infection in the upper airways, sustained or delayed IFN production in the lung has been proposed to fuel hyper-inflammation in patients with severe COVID-19 (26). Thus, we analyzed the production of IFN-I following Poly dA:dT stimulation of PC-derived PBMCs.

We observed that treatment with Poly dA:dT for 5 hours did not alter the levels of IFN-α produced by PBMCs from PC patients who did not develop lung fibrosis-like changes (**Figure 2A**). In contrast, as observed for IL-1α, stimulation of circulating cells from lung fibrotic PC patients induced a significant increase of IFN-α (p=0.0056) (**Figure 2B**). Interestingly, AIM2 activation did not induce the release of IFN-β from PC-derived PBMCs, even though they were stratified according to with or without fibrosis-like changes (**Supplementary Figures 1A, B**). To investigate the molecular mechanism/s that underpin IFN-α release induced by AIM2 stimulation, we treated fibrotic PC-derived PBMCs with Poly dA:dT in the presence of a caspase-1 or caspase-4 inhibitor, YVAD and ICH2, respectively. Pharmacological inhibition of both caspase-1 or caspase-4 reduced IFN-α levels after Poly dA:dT challenge (**Figure 2C**, p=0.0035 and p=0.0012, respectively) showing that there was a direct effect of both canonical and non-canonical AIM2 inflammasome pathways on the release of this cytokine. However, inhibition of cGAS with G140 did not alter IFN-α release from fibrotic PC-derived PBMCs although a trend was observed (**Figure 2D**). Similarly, the inhibition of STING with H151 did not statistically alter IFN-α levels (**Figure 2E**).

These results show that the release of IFN-α from PBMCs of PC patients with fibrotic-like changes was associated with canonical caspase-1-dependent, and non-canonical caspase-4-dependent, AIM2 inflammasome pathway activation.

### AIM2 Activation in PBMCs From PC Patients Did Not Induce Other IL-1-Type Cytokines

The cytokines of the IL-1-family (i.e., IL-1α, IL-1β, IL-18 and IL-33) play a significant role in inflammatory processes and their secretion is strictly dependent on multimeric inflammasome complexes (13).

**Figure 3:**
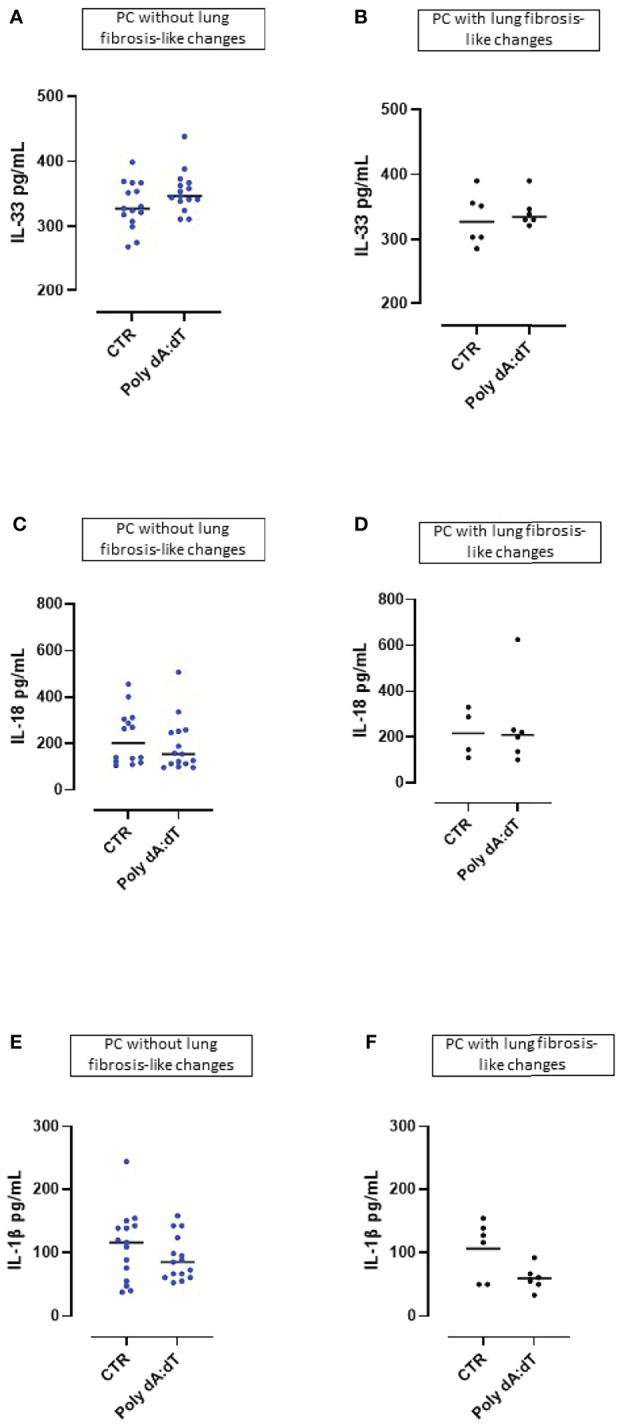
AIM2 receptor activation did not induced the release of inflammasome-associated IL-1-like cytokines from Post-COVID-19 patients with or without fibrosis. Stimulation with Poly dA:dT (1 μg/mL) for 5 hours did not induce the release of **(A, B)** IL-33, **(C, D)** IL-18 or **(E, F)** IL-1β from Post-COVID-19 (PC) PBMCs from patients without **(A, C, E)** or with **(B, D, F)** signs of lung fibrosis.

We found that challenge with Poly dA:dT did not induce IL-33 release from PBMCs obtained from either patients without lung fibrosis-like (**Figure 3A**) or with lung fibrosis-like changes (**Figure 3B**). Similarly, stimulation of the AIM2 receptor with Poly dA:dT for 5 hours did not lead to the release of IL-18 from PBMCs from PC patients without (**Figure 3C**) or with signs of lung fibrosis (**Figure 3D**). Importantly, stimulation of AIM2 responses in PBMCs obtained from both non-fibrotic (**Figure 3E**) or fibrotic (**Figure 3F**) PC patients, did not induce increases of IL-1β, a cytokine which is elevated in COVID-19 and correlates with disease symptoms (1).

These results indicate that PBMCs from PC patients were not susceptible to cytosolic dsDNA stimulation induced over-production of inflammasome-associated IL-1-like cytokines (IL-33, IL-18, IL-1β).

### AIM2 Inflammasome Triggering Induced the Release of TGF-β From Human PBMCs From PC Patients With Signs of Lung Fibrosis

In our previous study, we found that PC patients with GGO highlighted by chest CT scan were characterized by higher plasma levels of TGF-β, which is a well-known pro-fibrotic cytokine (11).

**Figure 4:**
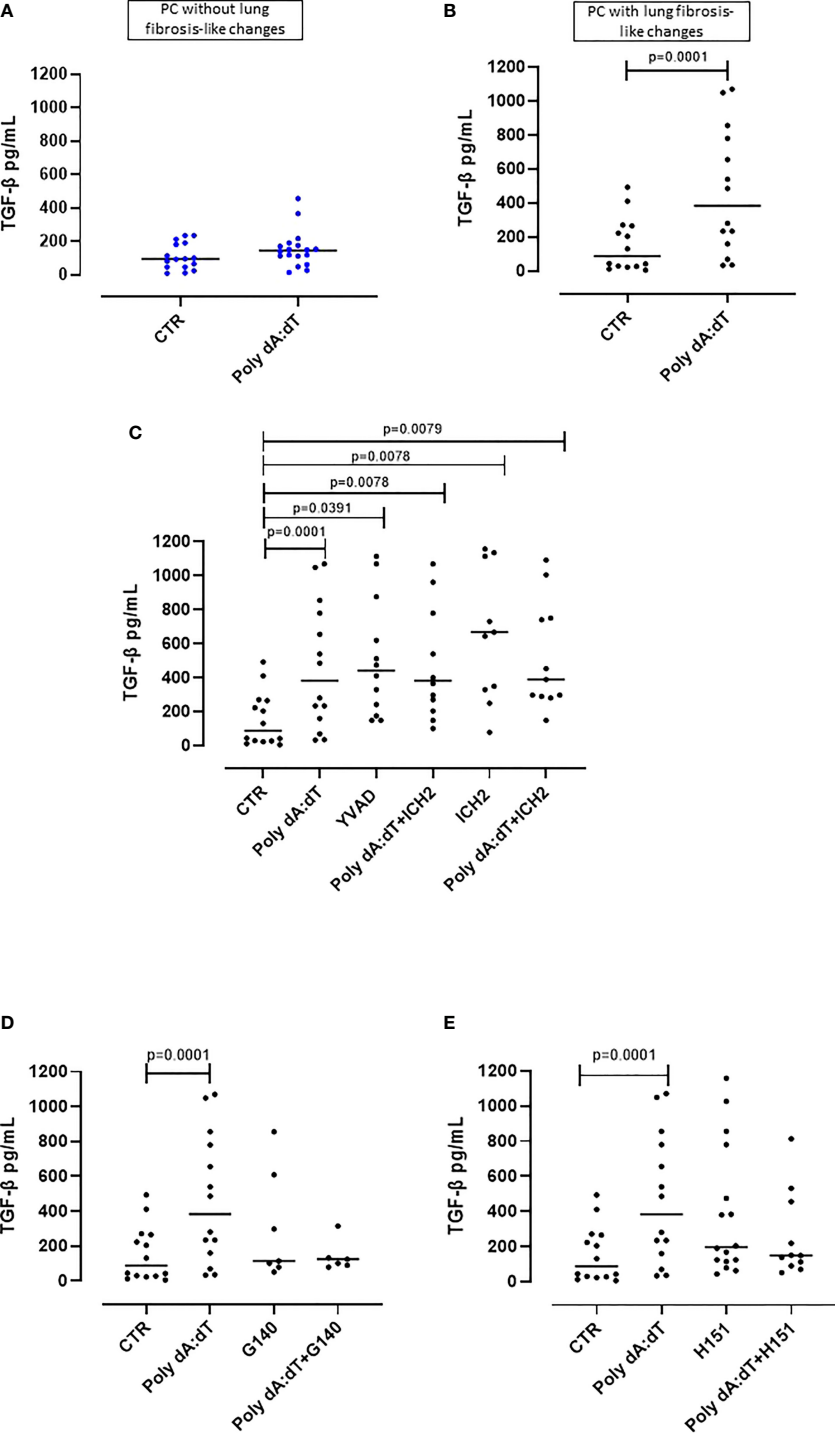
AIM2 receptor activation induced pro-fibrotic TGF-β release from PBMCs from Post-COVID-19 patients with fibrosis. **(A)** Stimulation of AIM2 in PBMCs from Post-COVID-19 (PC) patients without signs of fibrosis did not increase the release of TGF-β. **(B)** In stark contrast, stimulation of AIM2 in PBMCs from Post-COVID-19 (PC) patients with signs of fibrosis substantially increased the release of TGF-β levels. **(C)** Inhibition of caspase-1 and caspase-4 with Ac-YVAD-cmk (YVAD, 1 μg/mL) and ICH2 (ICH2, 6,4 μg/ml), respectively, did not reduce the release of TGF-β after Poly dA:dT stimulation. **(D)** Inhibition of cGAS or **(E)** STING with G140 (2 μg/ml) and H-151 (H151, 1 μg/mL), did not reduce the release of TGF-β after Poly dA:dT stimulation. Data are reported as median and represented as scatter dot plots. Statistical analysis was performed using the Wilcoxon matched-pairs signed rank test.

In this study, as previously observed, we found that the stimulation of the AIM2 receptor did not alter the release of TGF-β from PBMCs from PC patients with no sign of lung fibrosis (p=0.2015) (**Figure 4A**). In sharp contrast, PC patients with signs of lung fibrosis were susceptible to AIM2 triggering of TGF-β release (p=0.0168) (**Figure 4B**). To investigate the molecular mechanism/s associated with AIM2-dependent TGF-β release, we triggered AIM2 in fibrotic PC-derived PBMCs in the presence of caspase-1 or caspase-4 inhibitors, YVAD and ICH2. Pharmacological inhibition of either caspase-1 or caspase-4 did not reduce TGF-β levels after Poly dA:dT challenge (**Figure 4C**). This shows that there was not a direct effect of canonical caspase-1-dependent or non-canonical caspase-4-dependent activity of AIM2 inflammasomes on the release of this cytokine. As observed for IL-1α, to examine whether cGAS is an alternative cytosolic dsDNA sensor of Poly dA:dT, we evaluated the role of cGAS and STING inhibition with G140 and H151. We observed that neither cGAS (**Figure 4D**) nor STING (**Figure 4E**) inhibition significantly decreased Poly dA:dT-induced TGF-β release from fibrotic PC-derived PBMCs, however, there were non-statistically significant trends to decreases with both inhibitors (Poly dA:dT median: 383.9 pg/mL vs Poly dA:dT+G140 median: 124.6 pg/mL and vs Poly dA:dT+H151 median 149.7 pg/mL). If we compared treatment with G140 or H151, we did not observe any differences after cGAS (**Supplementary Figure 2A**) or STING inhibition (**Supplementary Figure 2B**).

Collectively, these results similarly to what we observed for IL-1α, suggest that there is an inflammasome-independent function of the AIM2 receptor that correlates with higher levels of the pro-fibrotic TGF-β from PBMCs from PC patients with signs of lung fibrosis. However, it should be noted that not all PC patient-derived PBMCs with signs of lung fibrosis had increased TGF-β (12/26, 46% of patients) after AIM2 stimulation.

### AIM2 Receptor Was Expressed at Higher Levels on Classical Monocytes From PC Patients With Signs of Lung Fibrosis

**Figure 5:**
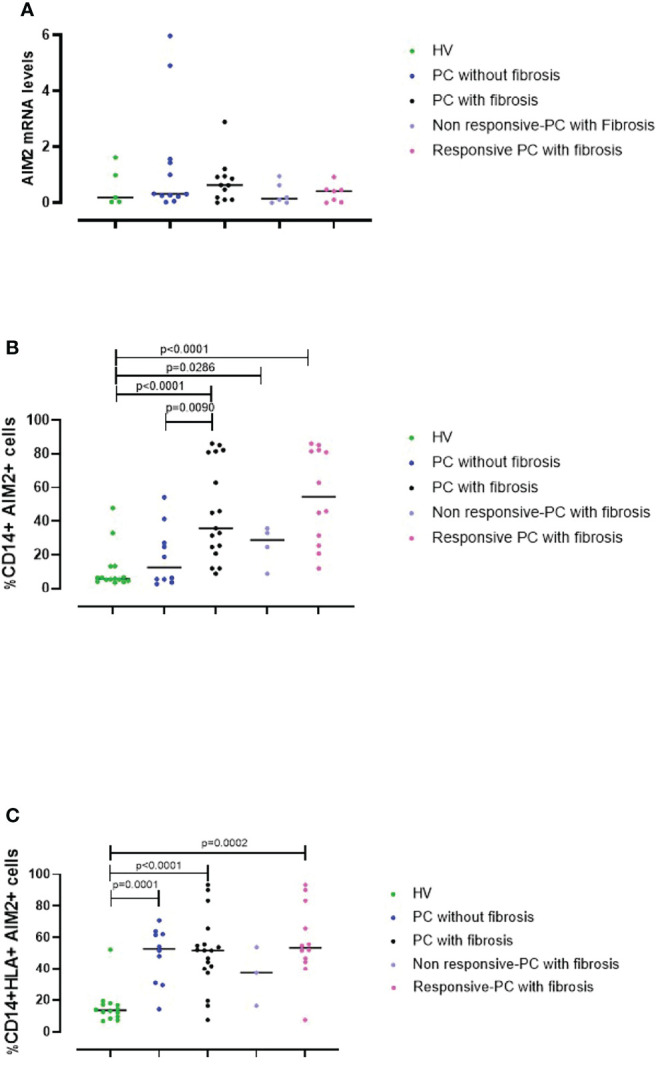
AIM2 expression in circulating cells. **(A)** The levels of AIM2 mRNA in PBMCs obtained from Healthy Vaccinated (HV) subjects (green dots), post-COVID-19 (PC) patients without (blue dots) and with fibrosis (black dots) were evaluated using RT-PCR. PC patients with fibrosis were stratified according to their responsiveness (release of cytokines) to Poly dA:dT stimulation, as non-responsive (light purple dots) or responsive (pink dots). PBMCs from the same groups were analyzed by flow cytometry for **(B)** CD14^+^AIM2^+^ and **(C)** CD14^+^HLA^+^AIM2^+^ expression. Data are reported as median and represented as scatter dot plots. Statistical analysis was performed using the Mann–Whitney U test.

To understand the role of AIM2 receptor in the PC syndrome, we evaluated the levels of AIM2 mRNA in PBMCs from HV controls and PC patients using RT-PCR. Results were verified with agarose gel electrophoresis, which showed a clear band at the expected molecular weight (158 bp) (**Supplementary Figure 3**). AIM2 mRNA levels were similar in HV (**Figure 5A**, green dots) and PC PBMCs, regardless of the absence or the presence of fibrotic-like changes (**Figure 5A**, blue and black dots, respectively). However, there was a trend to an increase in those with fibrotic-like changes. In addition, patients were also stratified according to the responsiveness to AIM2 activation. We considered patients PBMCs were non-responsive if they did not release cytokines after cytosolic dsDNA treatment, whereas as responsive PBMCs responded to Poly dA:dT stimulation by releasing at least one of the investigated cytokines (IL-1α, IFN-α, TGF-β). We observed no differences in AIM2 mRNA levels in these two sets of PBMCs, although there was a trend to an increase in responsive PBMCs from PC fibrosis donors (**Figure 5A**, light purple and pink dots, respectively). It should be pointed out that RT-PCR experiments were performed on RNA from total cells, which precludes the consideration of possible differences in AIM2 gene expression from specific immune cell populations in PBMCs.

To address this, we evaluated AIM2 protein levels using flow cytometry. This is because circulating monocytes, which represent ~20-40% of PBMCs are a major source of inflammatory mediators, and therefore may significantly contribute to the severity of COVID-19 (27). Thus, we evaluated AIM2 levels on both classical (CD14^+^ PBMCs) and inflammatory monocytes (CD14^+^HLA-DR^+^ PBMCs). By analyzing the circulating monocyte compartment, we found that fibrotic PC patients had significantly increased levels of AIM2 on classical monocytes compared to patients without fibrosis and the control HV group (**Figure 5B**, black vs green dots, p<0.0001). PC patients without fibrosis expressed similar levels of AIM2 receptor on CD14^+^ PBMCs as the HV group (**Figure 5B**, green vs blue dots, p=0.4047). Moreover, when PC patients with fibrosis were stratified according to responsiveness to Poly dA:dT stimulation, we found that the percentage of AIM2^+^CD14^+^ cells was higher in responsive compared to non-responsive PC patients although not significantly, mainly due to the small number of samples. Nevertheless, the levels of AIM2 in these subgroups were still higher than in HV subjects (**Figure 5B**, light purple and pink dots vs green dots, p=0.0286 and p<0.0001, respectively).

Interestingly, focusing on CD14^+^HLA^-^DR^+^ PBMCs, we observed that the percentage of inflammatory monocytes that expressed AIM2 was significantly higher in PC patients compared to HV subjects, both when we considered PC patients without signs of lung fibrosis (**Figure 5C**, blue vs green dots, p=0.0001) and with fibrotic-like changes (**Figure 5C**, black vs green dots, p<0.0001). It is noteworthy that PBMCs from patients without fibrosis, were not responsive to Poly dA:dT, showing impaired protective activity of AIM2 in the circulating cells of these patients. These data show that AIM2 expression was increased in inflammatory monocytes of PC patients, regardless of the presence of fibrotic-like changes. When we stratified fibrotic PC patients according to the responsiveness to Poly dA:dT treatment, we found that only responsive-PC patients had increased expression of AIM2 receptor compared to HV (**Figure 5C**, pink vs green dots, p=0.0002).

Together these data show that AIM2 expression in monocytes is associated with the development of fibrotic-like changes, and that the release of cytokines associated with AIM2 stimulation could exacerbate the pathogenesis of a fibrotic pattern in patients with PC syndrome.

## Discussion

Since the onset of the COVID-19 pandemic, a substantial proportion of patients who have been infected with SARS-CoV-2 continue to experience persistent symptoms after recovery. It has been reported that PC patients are susceptible to the development of pulmonary fibrosis as a consequence of lung damage and impaired gas exchange resulting from SARS-CoV-2-induced interstitial pneumonia and acute respiratory distress syndrome (ARDS) (6). It is also thought that the fibrotic lung damage that persists or develops after SARS-CoV-2 infection could correlate with excessive cytokine production. This ‘cytokine storm’ exacerbates inflammatory responses in the respiratory tract leading to the deposition of matrix components (7). In support of the role of the cytokine storm as a major pathogenetic mechanism associated with the establishment of PC-associated lung fibrosis, in our previous study we found that PC patients with GGO on chest CT scan were characterized by higher plasma levels of IL-1α and TGF-β, but not of IFN-β, compared to healthy and vaccinated subjects (11). In this study, we investigated the cellular source of cytokines which likely contribute to the pathogenesis of pulmonary fibrosis in PC patients.

We found that the stimulation of AIM2 led to IL-1α, IFN-α and TGF-β release from fibrosis-associated circulating cells from PC patients. Indeed, AIM2 activation led to the release of IL-1α and TGF-β through an inflammasome-independent pathway, whereas IFN-α release was caspase-1- and caspase-4-dependent. To date, little is known of the role of inflammasomes in PC syndrome, however, their activation has been suggested to be a major driver of severe COVID-19. Emerging evidence demonstrates that SARS-CoV-2 N protein interacts with NLRP3, leading to the formation of NLRP3-ASC complexes and the ensuing production of pro-inflammatory cytokines responsible for hyper-inflammation and lung injury in infected patients (15). Moreover, Rodrigues et al. (28), found that NLRP3 inflammasomes were active in postmortem lung tissues of deceased COVID-19 patients, and in serum samples and PBMCs collected from COVID-19 patients (28). In our study we found that PC PBMCs did not respond to NLRP3 triggering by LPS ± ATP in terms of pro-inflammatory cytokine release (data not shown), suggesting that these responses are impaired in PC. Moreover, to our knowledge, no studies have investigated the role of inflammasomes in the pathogenesis of fibrotic-like changes in PC conditions. Based on the similarities in terms of GGO observed at the chest CT scan and other common features of fibrosis between PC syndrome and IPF (6–8), we could speculate that as we observed for IPF (9), NLRP3 is not involved, but rather the stimulation of AIM2 in circulating monocytes could be involved in lung fibrosis-like changes in PC patients. In support of this, several studies have demonstrated that monocytes may be important drivers of severe COVID-19 (29, 30), and that they play major roles in initiating profibrotic responses by infiltrating the injured area and promoting tissue remodeling through the modulation of pro-fibrotic genes (31). In this context, we found that both CD14^+^ and CD14^+^HLA-DR^+^ PBMCs from PC patients with signs of lung fibrosis expressed higher levels of AIM2 and confirmed the release of IL-1α and TGF-β after its triggering. In contrast, AIM2 expression on circulating CD14^+^ PBMCs from PC patients without signs of lung fibrosis had much lower expression of AIM2 and lower levels of cytokine release. However, it has to point out that other authors (32) state that monocytes have no or low levels of AIM2, whereas CD27+ B cells express AIM2. In this regard, we were not able to detect circulating AIM2 positive B cells, most likely because of the pathology considered. Thus, we consider that AIM2 activation that leads to IL-1α, IFN-α and TGF-β release is likely to be involved in the establishment of lung fibrosis in PC syndrome. In support of our data, others have found that severe COVID-19 positively correlated with the levels of the soluble pro-fibrotic TGF-β (33). In addition, our previous work focused on plasma biomarkers, identified high levels of IL-1α and TGF-β in PC patients with fibrosis (11). In this study we identified a cellular source that releases these cytokines upon AIM2 inflammasome activation, that we previously defined as pro-fibrogenic (9).

AIM2 is widely known as a cytosolic receptor that recognizes dsDNA, initiates the oligomerization of ASC leading to the assembly of inflammasome complexes and triggering of an inflammatory cascade. However, recently, an inflammasome-independent function of AIM2 was suggested that occurs through novel crosstalk between AIM2 and cGAS, another cytoplasmic dsDNA sensor (25). It was suggested that dsDNA could induce inflammasome activation through an AIM2-independent cGAS-STING-NLRP3 pathway in human myeloid cells. According to our results, we found that the AIM2 inflammasome-dependent pathway was involved in the release of IFN-α from PC patients with signs of lung fibrosis. IFN-α is a member of the larger IFN family of cytokines that has pleiotropic roles in the regulation of both innate and adaptive immune responses (34). Much evidence suggests a role for a deficient type I IFN response to SARS-CoV-2 infection in COVID-19 progression (26), suggesting that SARS-CoV-2 infection hampers IFN-responses (10). A recent prospective observational study, evaluating IFN-α levels in a cohort of hospitalized COVID-19 patients with respiratory failure highlighted that increases in blood IFN-α levels were directly associated with improvement in COVID-19 disease severity and greater survival (11). Although the involvement of IFN-α in the pathogenesis of fibrotic-like changes in PC patients remained unexplored at that time, it was suggested that IFN-α could be a harmful factor in fibrotic lung diseases (35).

In contrast to what was observed for IFN-α, the release of IL-1α and TGF-β from PBMCs collected by PC patients who showed signs of lung fibrosis, after cytosolic dsDNA stimulation, was neither associated with caspase-1 nor caspase-4-dependent AIM2 inflammasome activation or to the cGAS-STING pathway. The mechanism of induction of these cytokines *via* AIM2 is still under investigation, but here we identify non-inflammasome related activity of AIM2. In addition, the cGAS-STING pathway is likely not involved in AIM2-induced IL-1α and TGF-β release.

Thus, although the precise role in disease burden remains unclear, the pronounced immune cell recruitment (36, 37) and release *via* AIM2 of pro-inflammatory (IL-1α and IFN-α) and pro-fibrotic (TGF-β) mediators from circulating monocytes are likely to be involved in lung fibrotic-like changes in PC conditions.

In conclusion, in the attempt to identify patients early who are at risk of developing fibrosis-like changes as COVID-19 sequelae and to define early intervention strategies, we consider that the release of IL-1α, IFN-α and TGF-β after AIM2 triggering and AIM2 expression on circulating monocytes could predict PC patients who are more likely to undergo fibrotic complications after COVID-19 recovery. Our study highlights a novel role of inflammasome-dependent and -independent AIM2 activity. This receptor mediates the release of pro-inflammatory and pro-fibrotic factors involved in PC-associated lung fibrosis, suggesting that AIM2 is an attractive molecular target that could help to early identify PC patients who are more likely to develop lung fibrosis and improve screening strategies.

## Data Availability Statement

The original contributions presented in the study are included in the article/**Supplementary Material**. Further inquiries can be directed to the corresponding author.

## Ethics Statement

The studies involving human participants were reviewed and approved by Ethical Board from Monaldi. The patients/participants provided their written informed consent to participate in this study.

## Author Contributions

CCo: Methodology, Writing – original draft; MT: Methodology; AMa: Methodology; AMo: Methodology; CCa: Methodology; CV: Visualization; PH: Supervision, Validation, Writing – review and editing; AV: Investigation, Visualization; AP: Investigation, Supervision; RS: Conceptualization, Data curation, Formal analysis, Investigation, Supervision, Writing – review and editing. All authors contributed to the article and approved the submitted version.

## Funding

RS was supported by FARB2020. CCo was supported by an Italian Research ministry fellowship. PH is funded by a Fellowship and grants from the National Health and Medical Research Council (NHMRC), Medical Research Future Fund of Australia (1175134), NSW RNA Production network, UTS and the Rainbow Foundation.

## Conflict of Interest

The authors declare that the research was conducted in the absence of any commercial or financial relationships that could be construed as a potential conflict of interest.

## Publisher’s Note

All claims expressed in this article are solely those of the authors and do not necessarily represent those of their affiliated organizations, or those of the publisher, the editors and the reviewers. Any product that may be evaluated in this article, or claim that may be made by its manufacturer, is not guaranteed or endorsed by the publisher.

## Supplementary Material

Supplementary Figure 1 Stimulation with Poly dA:dT (1 μg/mL) for 5 hours did not lead to the release of IFN-β from Post-COVID-19 (PC)-derived PBMCs, even when patients were stratified according to the **(A)** absence or **(B)** presence of signs of lung fibrosis on chest CT scan. Data are reported as median and represented as scatter dot plots. Statistical analysis was performed using the Mann–Whitney U test.

Supplementary Figure 2 Levels of TGF-β in matched pairs of PBMCs from Post-COVID-19 (PC) patients with signs of lung fibrosis after **(A)** cGAS or **(B)** STING inhibition with G140 (2 µg/mL) or H-151 (H151, 1 μg/mL), respectively, following AIM2 stimulation with Poly dA:dT (1 μg/mL). Data are represented as scattered dot plots. Statistical analysis was performed using the Wilcoxon t Test.

Supplementary Figure 3 Visualization of RT-PCR products using 2% agarose gel electrophoresis. RT-PCR product length of AIM2 mRNA was of 158 bp.
