# Supplementary figures and images for: Activation of the AIM2 Receptor in Circulating Cells of Post-COVID-19 Patients With Signs of Lung Fibrosis Is Associated With the Release of IL-1α, IFN-α and TGF-β
Source: Front Immunol. 2022 Jun 29;13:934264. doi: 10.3389/fimmu.2022.934264 (PMC9277546; doi:10.3389/fimmu.2022.934264)

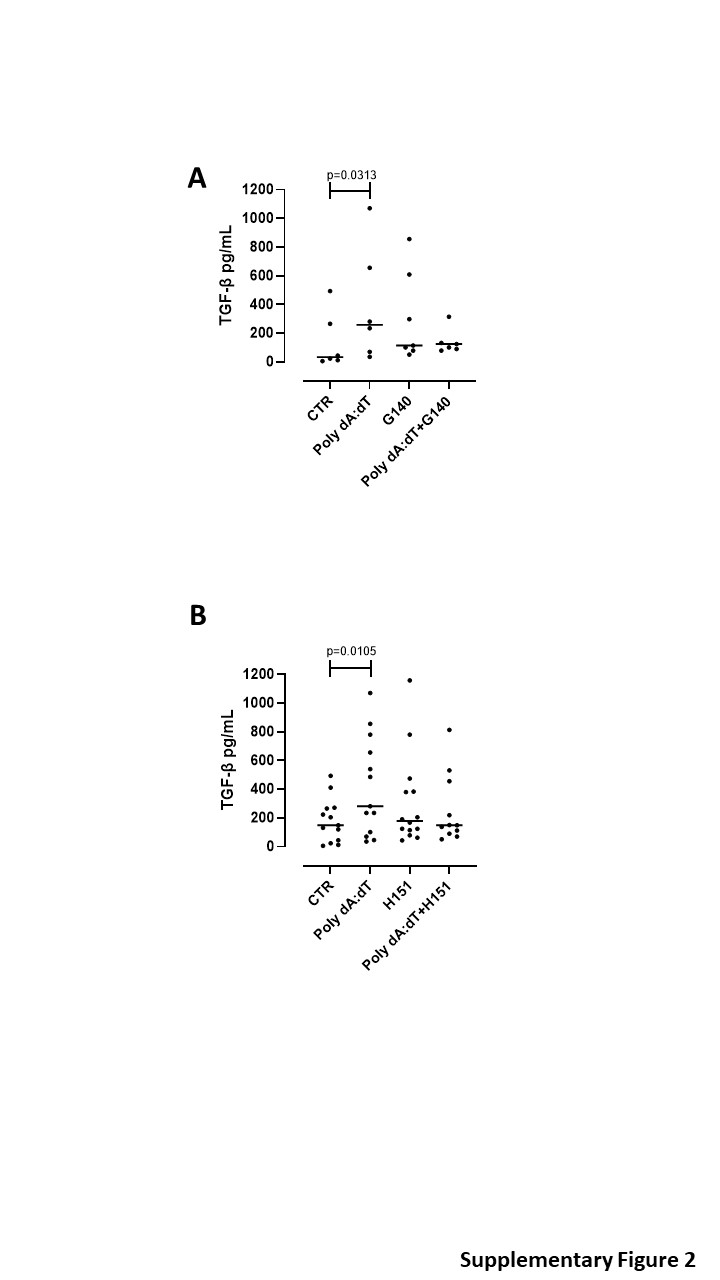

Supplement: Supplementary Figure 1 — Stimulation with Poly dA:dT (1 μg/mL) for 5 hours did not lead to the release of IFN-β from Post-COVID-19 (PC)-derived PBMCs, even when patients were stratified according to the (A) absence or (B) presence of signs of lung fibrosis on chest CT scan. Data are reported as median and represented as scatter dot plots. Statistical analysis was performed using the Mann–Whitney U test. [file Image_1.jpeg]

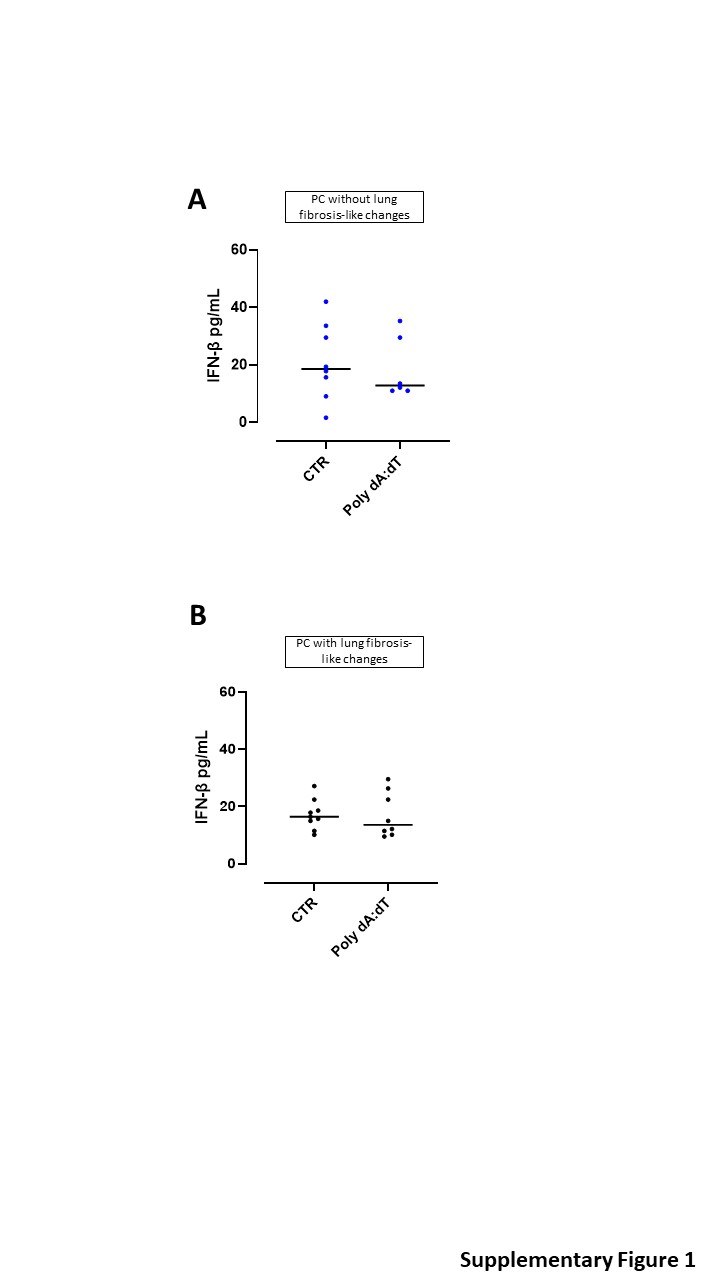

Supplement: Supplementary Figure 2 — Levels of TGF-β in matched pairs of PBMCs from Post-COVID-19 (PC) patients with signs of lung fibrosis after (A) cGAS or (B) STING inhibition with G140 (2 µg/mL) or H-151 (H151, 1 μg/mL), respectively, following AIM2 stimulation with Poly dA:dT (1 μg/mL). Data are represented as scattered dot plots. Statistical analysis was performed using the Wilcoxon t Test. [file Image_2.jpeg]

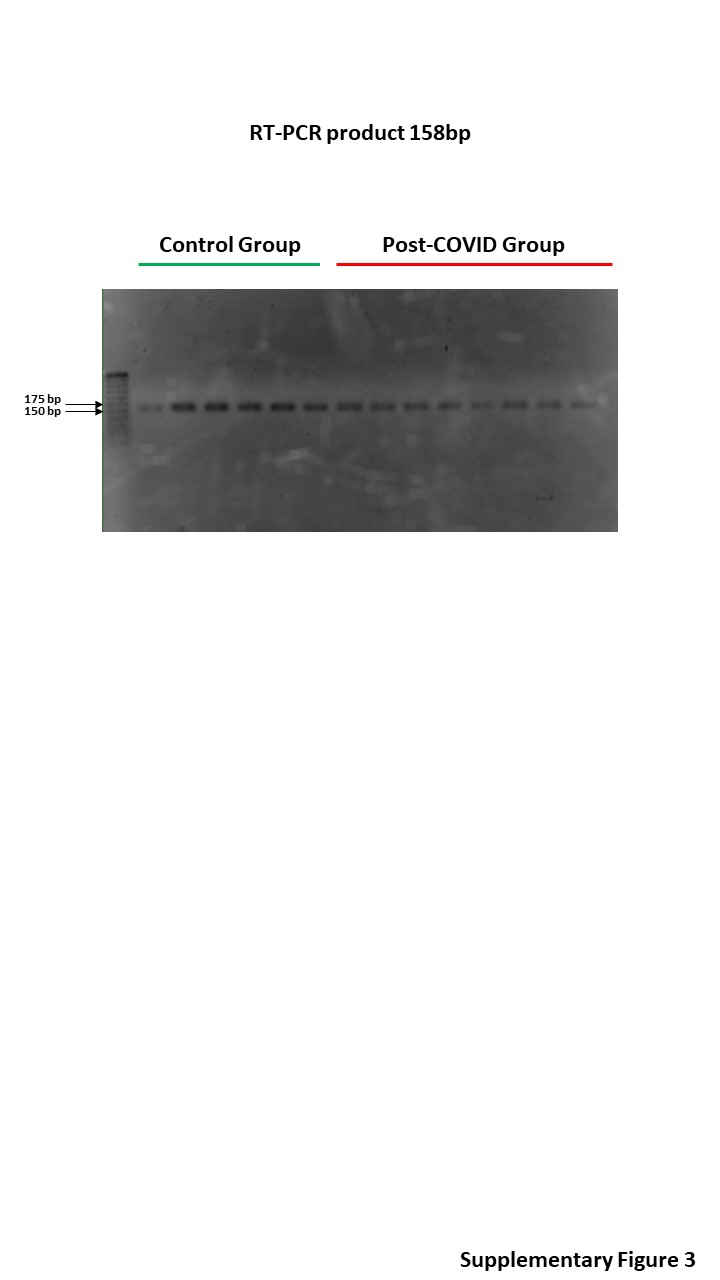

Supplement: Supplementary Figure 3 — Visualization of RT-PCR products using 2% agarose gel electrophoresis. RT-PCR product length of AIM2 mRNA was of 158 bp. [file Image_3.jpeg]
